# An Agreeable Surprise

**Published:** 1879-05

**Authors:** 


### AN AGREEABLE SURPRISE.

Editors, like other laborious people, have their dark hours of toil in which nothing encouraging is presented. Like others, however, an unexpected pleasurable incident breaks in upon them occasionally. While at our desk, on a chilly spring day, and coaxing our tired brain to the production of an acceptable editorial, our attention was attracted by the entrance of a boy, presenting a fine, gold-head walking cane, inscribed, "To Dr. J. G. W., 1879, V. H. T." With surprise and delight we accepted the present, and without any other evidence of the donor, recognized in the initials, "V. H. T.," our confrere, the popular obstetrician and gynecologist, in the Atlanta Medical College. The gift will ever be highly appreciated.

